# A quantitative method to measure geranylgeranyl diphosphate (GGPP) and geranylgeranyl monophosphate (GGP) in tomato (*Solanum lycopersicum*) fruit

**DOI:** 10.1186/s13007-023-01034-w

**Published:** 2023-06-07

**Authors:** Wayne Zita, Venkatasalam Shanmugabalaji, Miguel Ezquerro, Manuel Rodriguez-Concepcion, Felix Kessler, Gaetan Glauser

**Affiliations:** 1 Plant Physiology Laboratory, University of Neuchâtel, 2000 Neuchâtel, Switzerland; 2 Institute for Plant Molecular and Cell Biology (IBMCP), CSIC-Universitat Politècnica de València, 46022 Valencia, Spain; 3 Neuchâtel Platform of Analytical Chemistry, University of Neuchâtel, 2000 Neuchâtel, Switzerland

**Keywords:** Geranylgeranyl diphosphate, GGPP, Geranylgeranyl monophosphate, GGP, Geranylgeranyl diphosphate synthase, GGPPS, UHPLC–MS/MS, Isoprenoid, Carotenoid

## Abstract

**Background:**

Isoprenoids are a very large class of metabolites playing a key role in plant physiological processes such as growth, stress resistance, fruit flavor, and color. In chloroplasts and chromoplasts, the diterpene compound geranylgeranyl diphosphate (GGPP) is the metabolic precursor required for the biosynthesis of tocopherols, plastoquinones, phylloquinone, chlorophylls, and carotenoids. Despite its key role for the plant metabolism, reports on GGPP physiological concentrations in planta have been extremely scarce.

**Results:**

In this study, we developed a method to quantify GGPP and its hydrolysis product geranylgeranyl monophosphate (GGP) from tomato fruit, using ultra-high performance liquid chromatography coupled with tandem mass spectrometry (UHPLC–MS/MS). Quantification was done by external calibration and the method was validated in terms of specificity, precision, accuracy, and detection and quantitation limits. We further demonstrate the validity of our approach by analysing GGPP contents in the ripe fruits of wild-type tomatoes and mutants defective in GGPP production. Finally, we also show that the sample preparation is key to prevent GGPP hydrolysis and mitigate its conversion to GGP.

**Conclusion:**

Our study provides an efficient tool to investigate the metabolic fluxes required for GGPP supply and consumption in tomato fruit.

**Supplementary Information:**

The online version contains supplementary material available at 10.1186/s13007-023-01034-w.

## Background

**Fig. 1:**
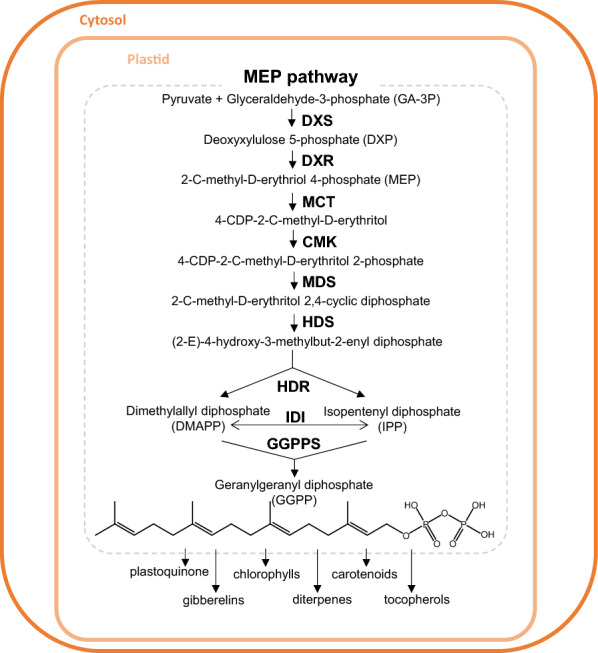
Schematic representation of the MEP pathway. Here, enzymes required for the biosynthesis of GGPP are represented. Enzymes: 1-deoxy-d-xylulose-5-phosphate synthase (DXS), 1-deoxy-d-xylulose-5-phosphate reductoisomerase (DXR), 4-(cytidine 5′-diphospho)-2-C-methyl-d-erythritol synthase (MCT), 4-(cytidine 5′-diphospho)-2-C-methyl-d-erythritol kinase (CMK), 2-C-methyl-d-erythritol 2,4-cyclodiphosphate synthase (MDS), 4-hydroxy-3-methylbut-2-enyl diphosphate synthase (HDS), 4-hydroxy-3-methylbut-2-enyl diphosphate reductase (HDR), isopentenyl diphosphate isomerase (IDI), geranylgeranyl diphosphate synthase (GGPPS)

In plants, isoprenoids (terpenes or terpenoids) are a large family of primary and secondary metabolites essential for a multitude of physiological and biological functions [1, 2]. Geranylgeranyl diphosphate (GGPP) is a C20 diterpene synthesised through the head-to-tail condensation of three isopentenyl diphosphate (IPP) groups and the head dimethylallyl diphosphate (DMAPP) by the geranylgeranyl diphosphate synthase (GGPPS) [3]. Plant cells synthesise IPP and DMAPP using the mevalonic acid (MVA) pathway in the cytosol or the methylerythritol 4-phosphate (MEP) pathway in plastids, where GGPP is most needed (Fig. 1) [2, 3]. Plastidial GGPP is a key component in plants as it is required for the biosynthesis of many photosynthesis-related terpenoids such as carotenoids, chlorophylls, tocopherols, phylloquinone, and plastoquinones [2–4]. MEP-derived GGPP is also used for the production of gibberellins and diterpenes (Fig. 1). In tomato (*Solanum lycopersicum*), five genes encode GGPPS-like enzymes. Among them, paralogs SlGGPPS1/SlG1 (Solyc11g011240), SlGGPPS2/SlG2 (Solyc04g079960) and SlGGPPS3/SlG3 (Solyc02g085700) have been localized in plastids and are required for GGPP biosynthesis [5, 6]. A recent study concluded that SlG2 and SlG3 are the main isoforms supplying GGPP in shoot tissues. Consistently, ripe fruit from *slg2* and *slg3* tomato knockouts showed decreased levels of lycopene, the red carotenoid that gives the characteristic colour to tomatoes [7].

Despite its central role in plants and other organisms, there is no commonly accepted method to measure GGPP at physiological levels. GGPP measurement represents a challenge due to its amphiphilic nature [8]. GGPP is not volatile, which makes its direct analysis by gas chromatography or gas chromatography–mass spectrometry impossible. In addition, the presence of phosphate groups complicates separation by conventional reversed-phase HPLC methods and may require ion-pairing chromatography or pre-column derivatization of the sample [9]. In addition, GGPP as an essential biosynthetic precursor in plant tissues is rapidly converted by enzymes to downstream secondary metabolites, complicating its detection. For these reasons, in most studies, instead of directly analysing GGPP, researchers have measured its downstream products such as carotenoids, quinones or other derived terpenoids [5, 6, 10, 11]. While some studies have proposed methods to detect GGPP in human plasma and cells using HPLC–MS or HPLC-fluorescence detection [12–14], we are unaware of any validated method able to measure GGPP physiological levels in plants. McCaskill et Croteau reported a complex and time-consuming procedure for the isolation and quantification of radiolabelled intermediates of the MVA pathway by ion-pairing chromatography coupled to radiodetection [15]. Recently, Ma et al. reported endogenous levels of GGPP in Arabidopsis leaves and inflorescences by UHPLC–MS/MS, however information on the methodology employed was limited [16].

In this work, we developed a simple but efficient method to measure GGPP and its hydrolysis product GGP in tomato fruit. The method is based on a single extraction step after quenching and lyophilisation of the fruits followed by reversed-phase UHPLC–MS/MS at alkaline pH. The method was validated according to standard guidelines and applied to the analysis of wild-type and GGPPS-defective *slg2* and *slg3* tomato fruits.

## Results and discussion

### Optimisation of HPLC–MS/MS conditions

**Fig. 2:**
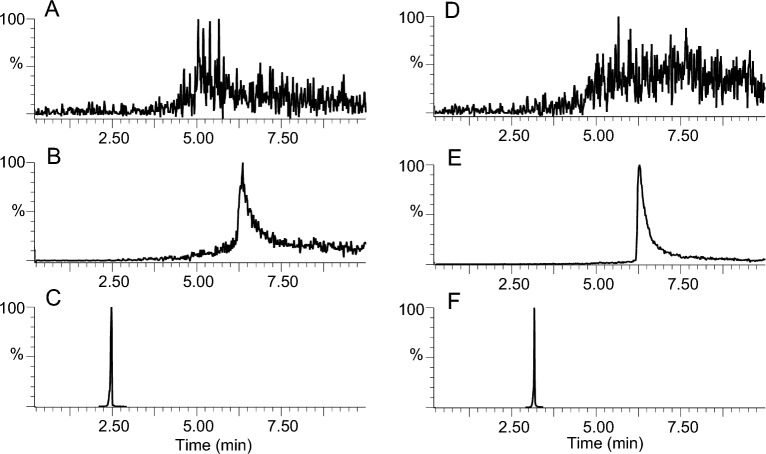
Chromatograms for GGPP and GGP at different pHs using an ethylene-bridged C18 column. **A**, **D** Chromatograms for GGPP and GGP at pH 2.7 (formic acid 0.05%); **B**, **E** chromatograms for GGPP and GGP at pH 5.8 (acetic acid 0.01% + 20 mM ammonium acetate); **C**, **F** chromatograms for GGPP and GGP at pH 10.0 (ammonia 0.05%). The organic mobile phase was acetonitrile in all cases

**Fig. 3:**
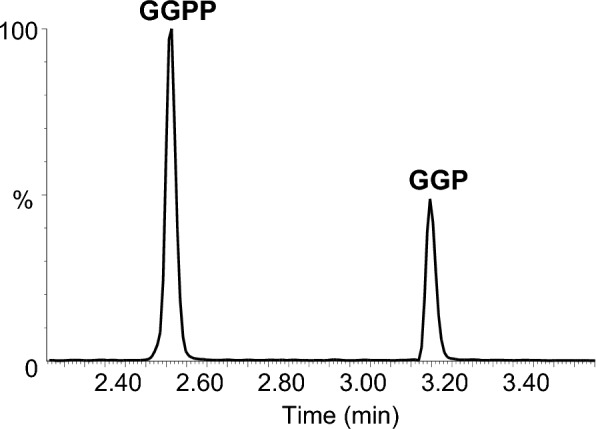
Representative chromatograms for standard solutions of GGPP and GGP. The concentrations of GGPP and GGP were 10 ng/mL and 1 ng/mL, respectively, both in acetonitrile:water (50:50, v/v). The MRM transition was *m/z* 369.2 > 79.0 for both molecules. A gradient of H_2_O + 0.05% NH_4_OH and acetonitrile + 0.05% NH_4_OH was applied

Due to the concomitant presence of a hydrophilic head (phosphate groups) and a lipophilic tail on their structures, there is no clearly defined scheme for the chromatographic separation of GGPP and GGP. Our aim was to find appropriate separation conditions without the need for ion-pairing or derivatisation agents, which may reduce detection sensitivity and lengthen the extraction process. During our trials, we found that hydrophilic interaction chromatography (HILIC) was not conclusive and thus focused on reverse-phase chromatography. Using an ethylene-bridged C18 column with a wide pH range, we observed a very strong dependency on the pH: at pH 2.7, using 0.05% formic acid as an additive in the aqueous mobile phase, the peaks for GGPP and GGP were extremely wide and almost indistinguishable from the baseline (Fig. 2A, D). By increasing the pH to 5.8 using a combination of 20 mM ammonium acetate and 0.01% acetic acid, the peak shapes improved but were still not acceptable to allow reliable quantitation (Fig. 2B, E). At pH 10.0, using 0.05% NH_4_OH as additive, the peaks became much thinner, although a slight fronting persisted (Fig. 2C, F). Adding 0.05% NH_4_OH to both aqueous and organic phases minimized peak fronting and enabled sharp and symmetrical peaks for both GGPP (RT 2.56 min, composition at elution 22.6%) and GGP (RT 3.20 min, composition at elution 28.4%) (Fig. 3). Acetonitrile as an organic solvent gave narrower peaks and slightly higher signal-to-noise ratios than methanol and was therefore preferred. The effect of the injection volume was also investigated. Using an injection solvent composed of 50–80% ACN, we found that peak distortion started to occur above 2 μL injections. We thus selected an injection volume of 2 μL as the best compromise between peak shape and sensitivity. Noteworthily, these conditions are valid for an Acquity UPLC system with a fixed loop injector in the partial loop with needle overfill mode. Preliminary assays using identical injection parameters on a flow-through needle (FTN) autosampler indeed revealed some peak distortion and further optimisation would be required when using this type of autosampler.

Mass spectrometric detection was performed on a triple quadrupole instrument (TQ-XS) of the last generation. We tested electrospray (ESI) and atmospheric pressure chemical ionization (APCI) sources, both in positive and negative ionisation modes. Not surprisingly, ESI and APCI positive ionisation gave no detectable peak. By contrast, the negative mode generated strong signals thanks to deprotonation of the phosphate group. Negative ESI was largely superior to APCI, with signals for GGPP and GGP approximately 10 and 15-fold higher, respectively. We tested different parameters of the ESI source, namely capillary voltage, source temperature, desolvation gas flow and temperature, nebulisation gas flow and cone gas flow. Only capillary voltage, desolvation gas temperature and cone gas flow had a significant impact on the signal-to-noise ratios, with optimal values at − 2 kV, 500 °C and 350 L/h, respectively. After having optimised source conditions, we determined MRM parameters. We found that GGPP readily lost a phosphate group in the MS source giving a prominent *m/z* 369.2 ion, corresponding to deprotonated GGP. The (M−H)^−^ ion at *m/z* 449 was also present, albeit at a lower intensity. We thus selected *m/z* 369.2 > 79.0 as the quantitative transition for both GGPP and GGP, with no risk of interference between them since the two molecules were well separated in the chromatographic dimension (Fig. 3). One and two additional qualitative transitions were found for GGP (*m/z* 369.2 > 97.0) and GGPP (*m/z* 369.2 > 97.0 and 449.2 > 97.0), respectively. Cone voltage and collision energies were tuned for maximal sensitivity and under optimized conditions, injections as low as 500 and 100 fg on column could be detected for GGPP and GGP, respectively.

### Sample preparation

**Fig. 4:**
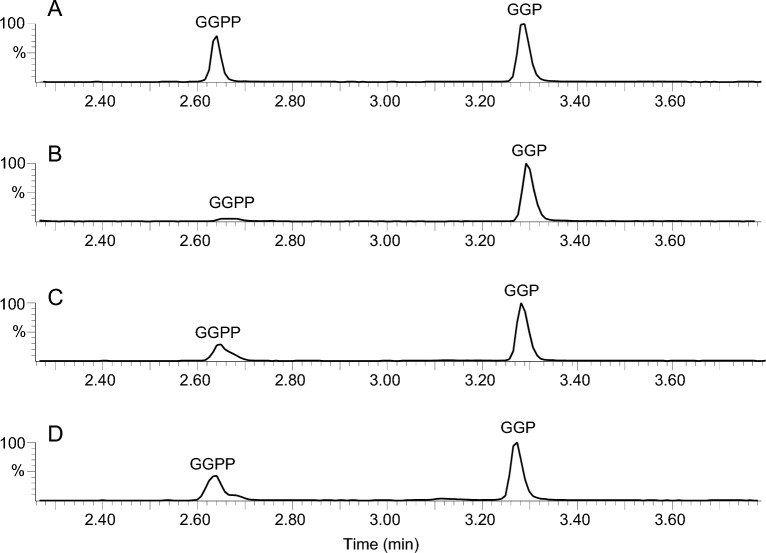
Chromatograms for wild-type tomato fruits extracted with different solvents or solvent mixtures. **A** Acetonitrile:water (80:20, v/v); **B** ethylacetate; **C** methanol; **D** methanol:water:NH_4_OH (70:30:0.05, v/v/v). For this experiment, fruits were not quenched in liquid N_2_ prior to extraction

Once we had an analytical method which was able detect trace levels of GGPP and GGP, we attempted to optimize their extraction from plant tissues. We used wild-type tomato fruits and first tested different extraction solvents: acetonitrile:water (80:20, v/v), ethylacetate (in this particular case with evaporation and reconstitution in acetonitrile:water (80:20, v/v) since ethylacetate was incompatible as an injection solvent), methanol and methanol:water:NH_4_OH (70:30:0.05, v/v/v) (Fig. 4). Unanticipatedly, results were very different, with acetonitrile:water (80:20, v/v) being the solvent which seemed to best preserve GGPP and ethylacetate the one which led to highest conversion to GGP (possibly due to the evaporation step). Furthermore, acetonitrile:water (80:20, v/v) was the solvent which provided the best extraction yield, determined as the sum of GGPP and GGP peaks. Based on these considerations, we selected acetonitrile:water (80:20, v/v) as the solvent of choice for GGPP extraction.

**Fig. 5:**
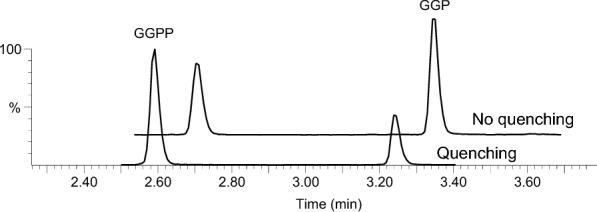
Chromatograms for wild-type tomato fruits submitted to quenching in liquid N_2_ immediately after sample collection or direct extraction in acetonitrile:water (80:20, v/v)

The observation that different solvents may lead to different rates of conversion from GGPP to GGP during extraction prompted us to test whether the typical sample preparation steps of quenching, drying and solvent evaporation could also have an impact on the GGPP/GGP ratio. Using acetonitrile:water (80:20, v/v) as extraction solvent in all cases, we observed that quenching the fresh fruits in liquid nitrogen prior to grinding and extraction had a significant effect in preserving GGPP (Fig. 5; *t*-test calculated on GGPP/GGP ratios, n = 3, p = 0.002). By contrast, after quenching there was no difference if frozen or lyophilised tissues were used (*t*-test, n = 3, p = 0.12). It should however be noted that, from a practical viewpoint, we found it much easier to grind lyophilised than fresh tissues using stainless steel beads in a tissue lyser. In addition, since GGPP and GGP have phosphate groups which may bind to metal cations, we tested the use of metal versus glass beads and found no difference between them. Finally, evaporation at 40 °C of the extraction solvent followed by reconstitution in the same solvent, namely acetonitrile:water (80:20, v/v), caused partial degradation of GGPP into GGP (*t*-test, n = 3, p = 0.02). Taken together, we thus recommend to quench the fruits as soon as they have been collected, lyophilise them as it makes subsequent steps easier, and avoid any evaporation during sample preparation to best preserve GGPP.

### Method calibration and validation

Spiking experiments with known concentrations of GGPP and GGP in plant extracts showed that matrix effects were negligible in tomato fruits. Therefore, the external calibration approach was selected and further evaluated during the method validation. Using linear calibration models, the r^2^ were > 0.99 for both GGPP and GGP and back-calculated concentrations were always within ± 10% of the true concentrations (Additional file 1: Tables S1 and S2). Specificity was excellent in fruit samples (Figs. 4 and 5). Precision and accuracy were determined at 4 different concentrations which were expected to cover the physiological concentrations in different mutants. RSD% (for precision) and deviations (for accuracy) always fell within 10% and 90–115%, respectively (Table 1). The fact that accuracy values were within acceptable ranges for both GGPP and GGP indicate that no significant conversion from GGPP to GGP occurred during the sample preparation process. The method’s limits of quantitation were 1 ng/mL (or 50 ng/g DW) and 0.17 ng/mL (or 8.5 ng/g DW) for GGPP and GGP, respectively. The limits of detection were 0.2 ng/mL and 0.03 ng/mL for GGPP and GGP, respectively. Taken together, these results demonstrate that the developed method is reliable and can be applied to the analysis of tomato fruits from various backgrounds.

**Table 1:** Precision and accuracy values obtained for GGPP and GGP in tomato fruits

	Precision (RSD%, n = 4)				Accuracy (%, n = 4)			
	C1	C2	C3	C4	C1 (%)	C2 (%)	C3 (%)	C4 (%)
GGPP	9.8	6.6	7.6	4.7	105	99	90	114
GGP	8.9	6.9	3.3	2.1	95	93	92	90

See “Methods” section for details about the actual concentrations corresponding to C1–C4

### Application to tomato fruits

**Fig. 6:**
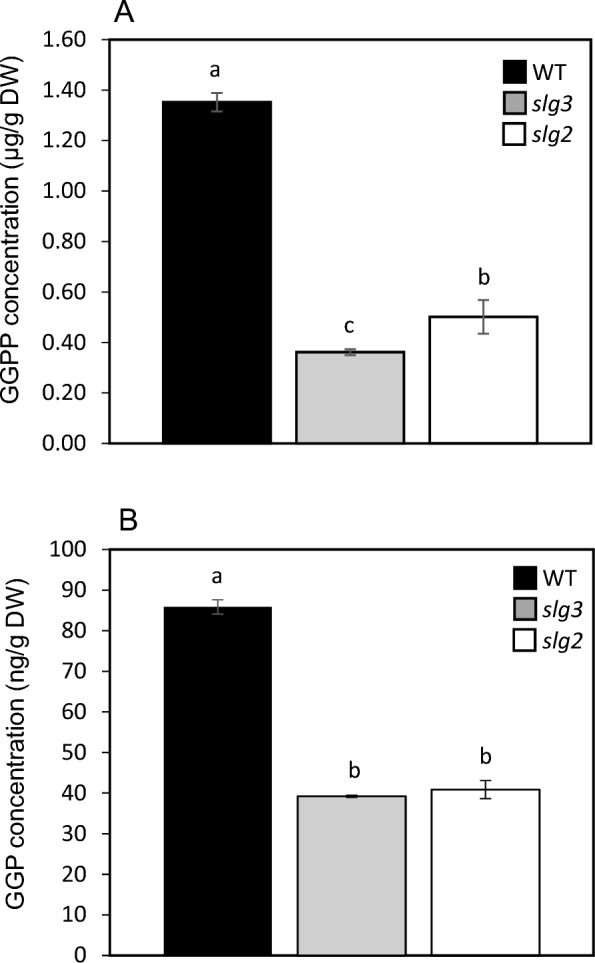
GGPP and GGP levels in *slg2* and *slg3* compared to WT tomato fruit. **A** GGPP absolute concentrations in WT, *slg2*, *slg3* B + 10 fruits; **B** GGP absolute concentrations in WT, *slg2*, *slg3* B + 10 fruits. Data are the means of three to four biological replicates (± SE). a–c significant differences between genotypes as detected by one-way ANOVA followed by post-hoc tests (p < 0.05)

To further validate our approach, we applied the developed method to the analysis of fruits from three “Micro-Tom” tomato genotypes: WT and two knockout mutants which lack one functional GGPPS (*slg2* and *slg3*). Fruits from these lines were tagged in the plant at the breaker (B) stage, i.e. when the first symptoms of colour change due to chlorophyll loss and carotenoid accumulation were visually detected. Ten days later, all fruits had acquired the characteristic red colour of ripe fruit. At this point (B + 10), fruits were collected from the plant and pericarp samples were snap-frozen in liquid nitrogen for subsequent lyophilisation. Levels of GGPP and GGP were significantly reduced in lyophilised fruit samples from *slg2* and *slg3* samples as compared to the WT (Fig. 6). Regarding GGPP levels specifically, WT, *slg2* and *slg3* contained 1.35, 0.50 and 0.36 µg/g DW, respectively. One-way ANOVA revealed significant differences between genotypes (F_2,7_ = 181.77, p < 0.001). Moreover, a Holm-Sidak post-hoc test showed that the levels of GGPP were significantly higher in *slg2* than in *slg3* (p = 0.037). This is consistent with the reported phenotype of carotenoid accumulation in these mutants, as described by Barja et al. [7]. These findings are interesting as they shed light on the role of different GGPPS paralogs in tomato fruit ripening [7]. Our results hence confirm the predominant role of SlG3 for the synthesis of GGPP required for the burst of carotenoids that changes the fruit colour from green to red when ripe. GGP levels were much lower than those of GGPP, with 0.087, 0.041 and 0.039 µg/g DW in the WT, *slg2* and *slg3*, respectively. This represents less than 10% of the actual GGPP levels and confirms that our method is gentle enough to prevent GGPP hydrolysis to a large extent.

## Conclusion

Our study establishes appropriate separation and detection conditions for GGPP and GGP without the need for ion-pairing or derivatization agents which may impact sensitivity and lengthen the extraction process. We show that reversed-phase chromatography at alkaline pH coupled to tandem mass spectrometry provides good peak shapes and sufficient sensitivity to enable the precise and accurate quantification of low levels of GGPP and GGP *in planta*. We applied our method to the analysis of ripe fruit from wild-type tomato and two knockout mutants of the main plastid-localised GGPPS isoforms. As expected, levels in the mutants were significantly lower than in the wild-type, thereby confirming the validity of our approach. Our method will be useful to identify new proteins and enzyme functions in a panoply of GGPP-dependent isoprenoid biosynthetic pathways with a view to improving tomato fruit quality and nutrition. In addition, the method may be extended to the detection of other prenyldiphosphates from the terpenoid pathway such as GPP and FPP to monitor new biotechnological approaches for plant and fruit fortification.

## Methods

### Chemicals

GGPP and GGP were purchased from Echelon Biosciences and Larodan, respectively. For HPLC–MS analyses, milli-Q water, LC–MS grade acetonitrile from VWR, and LC–MS grade NH_4_OH solution (25%) from Merck were used. For sample preparation, HPLC grade acetonitrile from Merck (Supelco) was employed.

### Plants

Tomato used in this study was *Solanum lycopersicum*, cv. Micro-Tom. Three genetic backgrounds were used, the wild-type (WT), as well as the CRISPR mutant alleles *slg2-1* and *slg3-1* [7]. Plants were grown in a chamber under controlled conditions (14 h under white light—150 μmol m^−2^ s^−1^—at 25 ± 1 °C and 10 h in the dark at 22 ± 1 °C). Fruits were tagged at the breaker (B) stage, harvested at B + 10 and quenched immediately in liquid nitrogen according to [17]. The samples were then stored at − 80 °C until lyophilisation in a Labconco benchtop freeze-dryer.

### Sample preparation

GGPP and GGP were extracted from lyophilised tomato fruits pericarp. Twenty mg of dry tissues were ground with 3 stainless steel UFO-beads (3.5 mm diameter) in a 2.0 mL microcentrifuge tube. Then, fifty volumes of acetonitrile:water (80:20, v/v) were added and the mixture was shaken in a tissue lyser (TissueLyser LT, QIAGEN) with a frequency of 50 Hz for 5 min. The homogenate was sonicated at 60 Hz for 1 min and centrifuged at 16,000×*g* for 10 min at room temperature. After centrifugation, 200 μL of supernatant was collected and transferred to glass vials for HPLC–MS/MS analysis.

### HPLC–MS/MS conditions

GGPP and GGP analysis was performed on a binary pump Acquity UPLC connected to a TQ-XS triple quadrupole (Waters), both controlled by MassLynx 4.2 (Waters). An Acquity UPLC BEH C18 column (2.1 × 50 mm, 1.7 µm, Waters) was used for the separation. The flow rate was set to 0.4 mL/min. Mobile phases consisted of milli-Q water + 0.05% NH_4_OH (phase A) and acetonitrile (ACN) + 0.05% NH_4_OH (phase B). The gradient started at 5% phase B and increased linearly to 41% B in 4.0 min, then to 100% B in 0.5 min. At the end of the run, a 2.0 min wash at 100% B for 2.0 min followed by re-equilibration at 5% B for 2.0 min was implemented. The column was maintained at 25 °C. The injection volume was of 2 μL (fixed loop injector, partial loop with needle overfill mode) and the autosampler temperature was kept at 15 °C. The strong needle wash was a mix of ACN:H_2_O (90:10, v/v) and the weak needle wash a mix of ACN:H_2_O (10:90, v/v).

The mass spectrometer was operated in electrospray negative ionisation using a capillary voltage of − 2 kV, a source temperature of 150 °C, a desolvation temperature of 500 °C, a desolvation gas flow of 1000 L/h, a cone gas flow of 350 L/h, and a nebuliser gas flow of 7 bars. The StepWave was set to normal transmission values. The multiple reaction monitoring (MRM) mode was employed to maximize sensitivity. MRM transitions for both GGPP and GGP were *m/z* 369.2 > 79.0 (quantitative, Q) and *m/z* 369.2 > 97.0 (qualitative, q1). For GGPP, an additional qualitative transition (q2, *m/z* 449.2 > 79.0, q2) was set. Cone and collision energy voltages for Q, q1 and q2 transitions were 15 and 21 V, 15 and 19 V, and 10 and 20 V, respectively. The dwell time was fixed to 136 ms. The HPLC flow was diverted to the waste from 0.0 to 2.25 min, and from 3.8 min to the end of the run.

### Quantification and method validation

Quantification was done by external calibration using standard concentrations in acetonitrile:water (50:50, v/v) at 1, 2, 4, 8, 16 and 32 ng/mL for GGPP, and 0.125, 0.25, 0.5, 1, 2 and 4 ng/mL for GGP. A linear calibration with the origin excluded and weighted by 1/x was applied. The response function of the calibration curve was assessed by back-calculating the concentrations based on the linear model and accepting it if the deviation was within 15% for all calibration points. Selectivity was evaluated by analysing non-spiked samples and samples spiked with a mixture of GGPP and GGP at 15 and 3 ng/mL, respectively. Intra-day precision and accuracy were expressed as percentage of relative standard deviation (%RSD) and percentage of deviation from true values, respectively, and were determined from samples of the *slg3* mutant spiked at 1, 3, 6 and 12 ng/mL (respectively C1–C4) for GGPP and 0.17, 0.5, 1, and 2 ng/mL (respectively C1–C4) for GGP (n = 4 for each concentration). Since GGPP and GGP are constitutively present in plants, including the *slg3* mutant, unspiked samples were also analysed and their concentrations subtracted from those of the spiked samples. Limits of quantification (LOQ) were determined as the smallest spiked concentration which gave precision and accuracy values within 15% and 80–120%, respectively. The instrumental detection limit was evaluated on standard solutions which gave signal-to-noise ratios of 3. Data processing was performed in TargetLynx XS (Waters).

### Statistics

All data in this study were the means of two independent experiments and the result of three to four technical or biological replicates, except the comparison of extraction solvents for which only two technical replicates were performed. No data were excluded from the analysis. The results were analyzed and compared for statistical differences by a two-sample, unequal variance (heteroscedastic) Student’s t-test (Excel 2016), or by one-way ANOVA followed by Holm-Sidak post-hoc tests for pair-wise comparisons (SigmaPlot v.15).

## Supplementary Information

**Additional file 1: Table S1.** Response function for GGPP with calibration equation y = 274.40x − 46.08 (R = 0.9990). **Table S2.** Response function for GGP with calibration equation y = 1405.94x − 4.6 (R = 0.9990).

## Data Availability

The datasets used for the current study are available from the corresponding author on reasonable request.
