# Supplementary material for: A quantitative method to measure geranylgeranyl diphosphate (GGPP) and geranylgeranyl monophosphate (GGP) in tomato (Solanum lycopersicum) fruit
Source: Plant Methods. 2023 Jun 7;19:55. doi: 10.1186/s13007-023-01034-w (PMC10249317; doi:10.1186/s13007-023-01034-w)
Supplement: Supplementary file 1 — Additional file 1: Table S1. Response function for GGPP with calibration equation y = 274.40x − 46.08 (R = 0.9990). Table S2. Response function for GGP with calibration equation y = 1405.94x − 4.6 (R = 0.9990). [file 13007_2023_1034_MOESM1_ESM.pdf]

A quantitative method to measure geranylgeranyl diphosphate (GGPP) and geranylgeranyl monophosphate (GGP) in tomato (*Solanum lycopersicum*) fruit

Wayne Zita[1], Venkatasalam Shanmugabalaji[1], Miguel Ezquerro[2], Manuel Rodriguez-Concepcion[2], Felix Kessler[1], Gaetan Glauser[3*]

Affiliations:
1 Plant Physiology Laboratory, University of Neuchâtel, 2000 Neuchâtel, Switzerland
2 Institute for Plant Molecular and Cell Biology (IBMCP), CSIC-Universitat Politècnica de València, 46022 Valencia, Spain
3 Neuchâtel Platform of Analytical Chemistry, University of Neuchâtel, 2000 Neuchâtel, Switzerland

*Corresponding author: gaetan.glauser@unine.ch

ADDITIONAL FILE 1

Table S1: Response function for GGPP with calibration equation y = 274.40x – 46.08 (R^2 = 0.9990)

Standard concentration (ng/mL)	Peak area	Back-calculated concentration (ng/mL)	Deviation (%)
1	253.4	1.09	9.1
2	489.7	1.95	-2.4
4	1004.0	3.83	-4.3
8	2098.9	7.82	-2.3
16	4247.1	15.65	-2.2
32	8917.9	32.67	2.1

Table S2: Response function for GGP with calibration equation y = 1405.94x – 4.6 (R^2 = 0.9999)

Standard concentration (ng/mL)	Peak area	Back-calculated concentration (ng/mL)	Deviation (%)
0.125	177.0	0.129	3.4
0.25	362.7	0.261	4.5
0.5	694.1	0.497	-0.6
1	1378.5	0.984	-1.6
2	2808.0	2.001	0.0
4	5623.9	4.003	0.1
